# Proteomics-Based Disease Biomarkers

**DOI:** 10.1155/2011/894618

**Published:** 2011-10-30

**Authors:** David E. Misek, Tadashi Kondo, Mark W. Duncan

**Affiliations:** ^1^Department of Surgery, University of Michigan, Ann Arbor, MI 48109, USA; ^2^Division of Pharmacoproteomics, National Cancer Center Research Institute, Tokyo 104-0045, Japan; ^3^Division of Endocrinology, Metabolism and Diabetes, School of Medicine, University of Colorado, Aurora, CO 80045, USA; ^4^Obesity Research Center, College of Medicine, King Saud University, Riyadh, Saudi Arabia

Sequencing of the human genome has greatly impacted the proteomics-based analysis of disease by providing a framework for understanding the proteome of diseased cells, tissues, and biological fluids. Consequently, there is a growing interest in applying proteomics technologies to define protein pathways involved in various diseases, to identify new biomarkers that correlate with diseases, ideally in their early stages, and to accelerate the development of new therapeutic targets. However, disease-related proteomics applications require that we improve our ability to separate and characterize the components of complex protein mixtures in such a way as to boost both throughput and sensitivity. In response to these demands, the proteomics technologies have been improved markedly over recent years. Today, proteomics, in all its various forms, is proving to be invaluable to our understanding of the biochemistry of health and disease and will likely play a central role in the evolution of personalized medicine. In this special issue, we include reports of novel research findings together with several reviews that highlight advances in key areas. 

The first two papers of this special issue focus on lung cancer. The first paper, by H. C. Gong et al., addresses the profiling of receptor tyrosine kinase pathway activation and the role of key genetic mutations in human lung tumor cell lines and human lung tumors. The authors defined molecular pathways which may assist in development of targeted lung tumor therapies. Within the second paper, Q. Zhang et al. used proteomic profiling to delineate expression and subcellular localization of multiple forms of aldehyde dehydrogenase in lung adenocarcinoma cell lines. The next two papers focus on pancreatic cancer. The third paper, by R. S. Kwon and D. M. Simeone, reviews the use of protein-based biomarkers for the diagnosis of cystic tumors of the pancreas. The fourth paper, by M. Abulaizi et al., utilizes a three-step proteomic protocol (immunodepletion of abundant serum proteins, followed by fractionation by RP-HPLC and further separation by 2D-PAGE) to discover candidate early detection biomarkers of pancreatic cancer.

The next two papers focus on breast cancer, with the fifth paper, by D. E. Misek and E. H. Kim, reviewing the development of protein biomarkers for the early detection of breast cancer. The sixth paper, by J. He et al., addresses LC-MS/MS identification of protein biosignatures in breast tumors, as protein-based markers that correctly classify tumor subtypes and predict therapeutic response would be of great clinical utility in guiding patient treatment. The next two papers are both by M. S. Sabel et al., and focus on melanoma. The seventh paper reviews the use of proteomics for the discovery of new prognostic and predictive biomarkers. The eighth paper explores the clinical utility of serum autoantibodies that were detected in melanoma patients. The investigators profiled serum antibodies against melanoma-associated antigens to identify those that may predict nodal positivity, a widely accepted index of metastatic disease. 

The ninth paper, by E. H. Kim and D. E. Misek, reviews the use of glycoproteomics to identify cancer biomarkers. The tenth paper, by A. Vivekanandan-Giri et al., utilized glycoproteomics to identify novel urinary glycoprotein biomarkers of chronic kidney disease. The issue concludes with two papers that report on novel approaches and related considerations. The eleventh paper, by I. Kiyokawa et al., describes the development of a new surface coating for urinary collection tubes that minimizes the amount of urine protein adsorption onto the walls of the collection tube. Within the final paper of this special issue, T. Hagiwara et al. examine the utility of a solid-phase hexapeptide ligand library in combination with conventional plasma proteomics modalities for comprehensive profiling of intact plasma proteins. 

*David E. Misek* *David E. Misek* *Tadashi Kondo* *Tadashi Kondo* *Mark W. Duncan* *Mark W. Duncan*

